# Diseases of the Maxillary Bones and Their Periosteum

**Published:** 1894-06

**Authors:** Vida A. Latham

**Affiliations:** F.R.M.S.; Chicago, Ill.


### THE DENTAL REGISTER.

VOL. XLVIII.] JUNE, 1894. [No. 6.

### COMMUNICATIONS.

### Diseases of the Maxillary Bones and their Periosteum.

Read in the Section on Dental and Oral Surgery, at the Forty-fourth Annual Meeting of the American Medical Association.

BY VIDA A. LATHAM, F.R.M.S., D.D.S , CHICAGO, ILL.

[*Continued from May Number, page* 230.]

*Tumors, Neoplasms, Non-Malignant* —Fibromata of the jaws resemble fibromata of other parts, but they have two origins: the periosteal, springing generally from the alveolus, and indistinguishable except by its size from epulis, and the endosteal, which springs from the interior of the bone, and in the upper jaw generally makes its way into the antrum and nasal cavities, or in the lower jaw expands the inner and outer plates of compact bone. Fibromata seem to owe their origin, in many cases, to the irritation of decayed teeth, which may sometimes be found embedded in the growth or displaced by it. The growths produce absorption by pressure, and may in this way destroy a great deal of structure.

Epulides are of two kinds, fibrous and sarcomatous. The fibrous epulis starts from the periosteum of the jaw, and is covered by the epithelium of the gum. The growth consists of long fasciculi of white fibrous tissue, which have a radiating arrangement, in many cases from the point of growth ; the most noticeable feature is the length and slenderness of the fibrous fasciculi and their loose felted arrangement. Epulides depend for their hardness on the amount of fibrous tissue they contain.

*Angiomata* may also occur, and I know of one case growing from the posterior wall of the antrum, which was diagnosed as osteo-sarcoma by a most competent physician and surgeon. The true nature of the growth is shown here by two micro-photographs, taken from the specimen before it was hardened, and it will easily classify itself into a fibro-angioma, showing two distinct kinds of structure, the fibrous and the angiomatous, thus accounting for the excessive hemorrhage which occurred during the operation. It is well to remember that we may have both simple, cavernous, lymph-angioma and angio-sarcoma.

*Enchondromata* are less frequent in occurrence in the jaws than fibromata, and like them may for convenience be divided into periosteal and endosteal. The disease usually appears early in life, springing from the surface of either jaw or from the antrum or the interior of the lower jaw, and has a more steady and rapid growth than fibromata. In the case of the superior maxilla the growth is more tuberous, and is apt to send off processes into the fissures and cavities of the skull, giving rise to great deformity. Enchondromata as regards their micro-structure vary greatly, and we must prepare for many enigmas in studying them. Whenever chondroma is associated with sarcoma it seems to take an erratic course; when however, a pure chondroma is found, it is always more near the normal type of cartilage. All the chondromata are very liable to calcification, and they sometimes undergo a softening process and small cysts form in them.

*Osteomata* are a further step in the development described under chondromata. They are formed from newly-developed connective tissue, and it is a fact which distinguishes them from so-called ossified inflammatory products. After decalcification, under the microscope they resemble true bone, and, like it, can be divided into hard and cancellous. Many, however, vary a good deal from the normal type, especially the hard variety, which is sometimes extremely dense.

Osteomata have been described as primary growths, but are extremely rare. True bone is sometimes found in cartilaginous and fibrous tumors, and also in sarcoma as the myeloid variety. The cancellous osteoma is the simplest form, and due usually to a misplaced tooth; and the fact that numerous serious operations have been performed in these cases, should make every one extremely careful in their diagnosis. The growth is slow and reaches a large size, but when removed by section of the healthy bone it shows no tendency to recur.

Under the term, cystic sarcoma, many varieties of growths were classed, and by some authors termed a variety of epithelioma, (see Mr. Eve, in Erasmus Wilson Lectures for 1882), but they differ so completely from the ordinary form, as to rapidity and recurrence after removal, that as yet further observations must be made before any decision can be arrived at.

The largest and most dangerous class of growths met with are the *sarcomata*, divided into three classes by the shape of their cells: *a*, round; *b*, spindle; *c*, myeloid. And these have again a number of modifications and varieties. As the general features are so well known, little or no description will be given beyond a few points of interest which may occur in them. The spindle-celled sarcoma is quite frequent, forming many of the specimens so indiscriminately named "osteo-sarcoma." A point worthy of notice in the recurrent growths is the tendency to become softer with each recurrence, and the patient dies, worn out, with rarely secondary deposits in internal organs.

Round-celled sarcoma has unfortunately not been clearly defined in oral surgery until the progress in pathology became so marked, and even now it is often named wrong. It is still called encephaloid sarcoma of Cornil and Ranvier and others, when we to-day usually regard encephaloid not as of the sarcoma, but of the carcinoma group, as it originates from glandular epithelium. Many of the cases called medullary cancer of the jaw, belong to the class of round-celled sarcoma. Myeloid sarcoma, originally described by Paget, is very common, and found in connection with the alveolus, forming the so-called myeloid epulis, and also in the interior of the lower jaw. The growths vary enormously histologically. In the first place they get their name from the large cells they contain, which closely resemble those found in the marrow of bone; and usually are known by "their red color, resembling raw beef, and are named by Butlin, "a mixed celled sarcoma." In connection with the pure form of myeloid sarcoma we have myxomatous tissue and bony formation or ossification closely combined. The ossification is usually a peculiar shape, the material formed resembles decalcified bone, and contains no lime-salts. By using double and triple staining processes the different reactions in the normal formation can be made out very nicely, and the lime deposited by each individual cell; but in the sarcomatous form of ossification it so far has not been shown. The ossifying process must not be confounded with cases where the growing sarcoma has decalcified the bone in its immediate neighborhood; this is sometimes the case, especially in bones like the scapula, and portions of this in the middle of a sarcomatous growth will cut like fibrous tissue without any decalcifying process. Under the microscope this can readily be made out; the lacunæ in them are larger and often empty, and there are no osteoblasts on their free edges. That sarcoma has a decalcifying power is seen in China, where the jaws of horses are often attacked and so softened that you can cut through them with an ordinary knife, just like cutting a piece of cheese. Myeloid sarcoma also forms one kind of epulis, and care must be taken in making a diagnosis.

Besides these forms, we have alveolar sarcoma, which is a rare and unsatisfactorily described growth. By Wedl it is called "a fibrous form of cancer arising from bone," and should be included under cases hitherto given as scirrhus of bone, which is not strictly correct.

Fibro-sarcoma grows beneath the periosteum, and closely resembles fibroma.

Chondro-sarcoma is a mixed growth, and occurs in both jaws, and frequently leads to secondary deposits in the lungs.

Ossifying sarcoma has been classed for greater convenience under the enchondromata and osteomata and myeloid sarcoma.

Finally, we have to consider carcinoma, which for convenience has been classed as follows into two groups, according to the character of the cells from which they are formed:

Superficial epithelioma: squamous epithelioma.

Glandular epithelium: *a*, columnar epithelioma; *b*, scirrhus carcinoma; *c*, encephaloid carcinoma.

Carcinoma affecting the jaws is only epithelioma of the squamous and columnar form. The first variety developing primarily in the mucous membrane of the palate and gums; the columnar form beginning in the antrum or nose. The two forms rapidly invade all the tissues, even to the bones. When squamous epithelioma begins in the palate or gums it is very often not noticed, and its nature is often mistaken at first. Beginning as a small ragged ulcer, it is often attributed to decayed roots or secondary syphilis, etc., and only locally treated. Ulcer of the palate of an epitheliomatous character is more frequently attributed to tertiary syphilis, and even large gaps in the hard palate caused by epithelioma are supposed to be the result of a broken-down gumma. By involving the subjacent bone, necrosis is induced in the course of an epithelioma, and here again error may arise if the presence of bare bone be regarded as pathognomonic of necrosis, without considering the cause.

Much information and profit can be gained by consulting the following works, to which I owe much information, especially the valuable works of Prof. C. Heath:

BIBLIOGRAPHY.

Heath, C., Injuries and Diseases of the Jaws.
Heath, C., Lectures on Two Certain Diseases of the Jaws.
Heath, C., Dictionary of Surgery.
Butlin, On the Tongue.
Paget, Surgical Pathology.
Pepper, Surgical Pathology.
Ziegler, Pathological Anatomy.
Eve, Erasmus Wilson Lectures, 1882.
Sutton, J. Bland, Principles of Pathology.
Garretson, Oral Surgery.
Tomes, Dental Surgery.
Tomes, Comparative Anatomy.
Sutton, J. B., Trans. Odont. Soc., 1881, etc.
Wedl. Pathology of the Teeth.
Adams, Path. Soc. Trans., Vol. XII.
Hutchinson, Path. Soc. Trans., Vol. VIII., p. 380.
Salter, Dental Pathology and Surgery.
Salter, Guy's Hospital Reports, 1856.
Journal. Brit. Dent. Assoc. (Malassez.)
Dental Cosmos.
American Text-Book of Surgery.
Black, J. V., Dental Periosteum.

DISCUSSION.

Dr. J. S. Marshall said the paper was an excellent one, and the author deserved the thanks of the profession for preparing it. Discussing the paper, he said that it was often difficult to distinguish with certainty malignant growths in the jaws, but he thought it best in cases where there was any real doubt to cut away tissue, though it may be benign, rather than take the chance of letting a malignant growth increase, and so endanger the life of the patient.

The tumors most frequently seen are called epuloid, and he thought the one described by the essayist to have been of that character. These are not usually malignant, but they sometimes take on a malignant character. He could not account for the great swelling in connection with a tumor of this size. Dr. Latham had asked him about it, and he had told her he had not seen such a case; however, she had been informed by other dentists that it was common. His treatment would be the immediate and thorough removal of such a tumor for fear it would take on a malignant growth.

Dr. Taft asked how long it had been since the tooth was removed?

Dr. Latham answered: "In February; no other tissue was removed, the tooth came away quite easily, and the cavity healed up very nicely."

Dr. Taft said this emphasizes the danger of making more of a case than belongs to it. If the parts returned to a healthy state it would eliminate any idea of malignancy. In diagnosing and treating such cases, much attention should be paid to the physical characteristics of the patient. If his condition is generally good, it is safe to consider that the tumor is of a benign character, while a tumor of the same appearance and character in one whose system was diseased would probably take on a malignant character. He had been called upon to assist a physician to remove what the physicians called an epulis. To his surprise, the physician removed the cuspid and bicuspid teeth, then took a saw and cut out a notch including the sockets of the two teeth. His own treatment would have been to have considered the growth a little, insignificant, benign epulis, and he would have removed it with a pair of scissors. He spoke of several cases in his own practice of the kind, and said it was well not to be too hasty in pronouncing a growth of this character malignant. The growth, origin and causes should be studied, and the case kept under careful observation for some time.

Dr. Marshall said the only positive way to diagnose a tumor is by the microscope. When the tumor is large enough and of such a character that a piece can be cut from it with the scissors, he examines it himself, and has it examined by some one in whom he has confidence, and if it is pronounced malignant then he removes it, going deep enough to be sure to remove all the affected tissue. By prompt and thorough removal the life of the patient may be saved, but if operation is deferred until the disease has invaded the glands, the growths will return at the same, or at some other place, and the life of the patient will not be for long. He related the case of a patient, a man about sixty years of age, with an epuloid tumor between the first and second bicuspids on the right lower side. There was considerable ulceration, involving gums and cheek, almost to the corner of the mouth. On examination it was pronounced an epithelioma. He made a most thorough operation, removing three teeth, the alveolus down to the dental canal, and part of the cheek. The parts healed nicely; the wound in the cheek by first intention, and fifteen months having passed since the operation, there has been no recurrence of the growth. He, however, expected that if the man lived a few years it would recur, either where it was before or in some other place.

Dr. Taft said that the class of patients operated upon in general hospitals was so far below the class that a dentist or physician would have in his private practice, that it was not safe to accept what was true of one class as applying to the other. Hospital patients were, to a very large extent, little more than wrecks, their whole system broken down by poverty, vice, and disease; no vigor or recuperative power, and a disease which could be safely handled and cured under better circumstances was well nigh hopeless with them.

He spoke of a case in his practice of a girl with a tumor on her jaw as large as a pigeon's egg. Relief was sought because of the rapidity of the growth. Upon examination, Dr. Taft thought it not malignant. Two surgeons were consulted. The first one after a careful examination said he could not decide; the second after looking at it two minutes said it was malignant, and unless the whole side of the jaw was removed the girl would die of it. The decision arrived at, however, was to merely remove the tumor and carefully watch the case. Some time after the tumor returned and electrolysis was used, and it entirely healed and never returned. If the advice of the surgeon number two had been taken and the girl's jaw sawed away, she would have been mutilated for life. He spoke of the great importance of the dentist being able to judge of the character of such growths. They come under the notice of the general practitioner. The best manner of removing them when small, and of a favorable shape, was by a ligature. This is painless and saves what sometimes would be an alarming loss of blood. The patient should be carefully treated before and after the operation so as to get and keep the system in the best possible condition.

Dr. E. S. Talbot spoke of the growth of secondary dentine as shown in the case of which Dr. Latham spoke. These growths were difficult to diagnose, and they produce periostitis and neuralgia. They are most frequent in the pulp chamber and root canal, and neuralgia results. The only treatment is the removal of the tooth.

Dr. J. S. Marshall spoke of the formation of calcific deposits in the pulp chamber, and said that, as a rule, peripheral calcifications do not give any trouble, but the interstitial form always makes trouble. One reason that they are difficult to diagnose is that the patient can not place the seat of the trouble, though sometimes the tooth feels big.

Dr. A. E. Baldwin said that it was best when in doubt to treat an epuloid growth as if it was malignant, as it was better to go too deep than by taking the other course to endanger the life of the patient. We should, however, take advantage of the knowledge of other physicians when attainable, and above all other things fit ourselves to be able to discriminate by a wider and deeper course of study and research. The whole field of microscopy is opened by such a subject as this, and it is only the intense and rapid student that can take advantage of the facilities for learning that come to our hands. He spoke of a case of his own which he thought was a simple, harmless growth, but took advantage of the offer of a friendly microscopist to examine it, when it was found to be malignant, and so he operated accordingly.

Dr. Latham, in closing the discussion, said that there were some dentists who never looked further than the tooth they were working on, and she thought they should be more careful to examine the whole mouth and note every appearance of disease, thereby serving their patients better. She spoke of a case in her own practice where she had discovered an epithelioma, which would be removed by a surgeon. She thought that it was dangerous, however, for a dentist to undertake the treatment, by means of caustics or other remedies, of growths in the mouth unless they had sufficient knowledge to discriminate between harmless and malignant tumors.

